# Does Sars-Cov-2 threaten our dreams? Effect of quarantine on sleep quality and body mass index

**DOI:** 10.1186/s12967-020-02465-y

**Published:** 2020-08-18

**Authors:** Luigi Barrea, Gabriella Pugliese, Lydia Framondi, Rossana Di Matteo, Daniela Laudisio, Silvia Savastano, Annamaria Colao, Giovanna Muscogiuri

**Affiliations:** 1 Dipartimento di Medicina Clinica e Chirurgia, Unit of Endocrinology, Federico II University Medical School of Naples, Via Sergio Pansini 5, 80131 Naples, Italy; 2 Centro Italiano per la cura e il Benessere del Paziente con Obesità (C.I.B.O), Department of Clinical Medicine and Surgery, Endocrinology Unit, University Medical School of Naples, Via Sergio Pansini 5, 80131 Naples, Italy; 3 Cattedra Unesco “Educazione alla salute e allo sviluppo sostenibile”, University Federico II, Naples, Italy

**Keywords:** CoVID-19, Sars-Cov-2, Obesity, Quarantine, Sleep disturbance, Sleep quality, Smart-working, Nutritionist

## Abstract

**Background:**

*COVID 19*-*related* quarantine led to a sudden and radical lifestyle changes, in particular in eating habits. Objectives of the study were to investigate the effect of quarantine on sleep quality (SQ) and body mass index (BMI), and if change in SQ was related to working modalities.

**Materials:**

We enrolled 121 adults (age 44.9 ± 13.3 years and 35.5% males). Anthropometric parameters, working modalities and physical activity were studied. Sleep quality was evaluated by the Pittsburgh Sleep Quality Index (PSQI) questionnaire. At baseline, the enrolled subjects were assessed in outpatient clinic and after 40 days of quarantine/lockdown by phone interview.

**Results:**

Overall, 49.6% of the subjects were good sleepers (PSQI < 5) at the baseline and significantly decreased after quarantine (*p *< 0.001). In detail, sleep onset latency (*p *< 0.001), sleep efficiency (*p *= 0.03), sleep disturbances (*p *< 0.001), and daytime dysfunction (*p *< 0.001) significantly worsened. There was also a significant increase in BMI values in normal weight (*p *= 0.023), in subjects grade I (*p *= 0.027) and II obesity (*p *= 0.020). In all cohort, physical activity was significantly decreased (*p *= 0.004). However, analyzing the data according gender difference, males significantly decreased physical activity as well as females in which there was only a trend without reaching statistical significance (53.5% vs 25.6%; *p *= 0.015 and 50.0% vs 35.9%, *p *= 0.106; in males and females, respectively). In addition, smart working activity resulted in a significant worsening of SQ, particularly in males (*p *< 0.001).

**Conclusions:**

Quarantine was associated to a worsening of SQ, particularly in males doing smart working, and to an increase in BMI values.

## Background

Recently the whole world experienced the enormous stress of the pandemic of severe acute respiratory syndrome coronavirus 2 (SARS-CoV-2) that began in Wuhan, Hubei, China in late 2019 and it has been renamed CoVID-19 by the World Health Organization on February 2020 [1]. In order to reduce the spread of the virus and to reduce the impact of a huge number of infected subjects on medical resources, at the beginning of March 2020 Italy went into emergency quarantine, with stringent containment measures on the entire national territory [2]. These containment measures enacted through the *#iorestoacasa* decree [2] that leads to a sudden and radical lifestyle changes, in particular in eating habits. Quarantine leads to staying at home with smart-working and the reduction of the outdoors physical activity or in the gym. Of interest, quarantine could be also associated with an unhealthy diet, poor in fresh foods as fruit and vegetables and rich in processed food due to the limitation on daily shopping. It is known that an unhealthy diet is associated with obesity and sleep disturbance [3]. In particular poor diet quality characterized by high fat and low fiber intake has been reported to be associated to sleep disturbance [4, 5]. In addition to fat, carbohydrates-rich meal in the evening has been reported to results in an increase of core body temperature ad heart rate and in a reduction of nocturnal secretion of melatonin within the 8 h after the end of the meal consumption in healthy normal weight subjects [6, 7]. Most of the time carbohydrates, in particular sugars, are identified as ‘comfort foods’ due to their property of increasing serotonin production that in turn has a positive effect on mood [8]. In a sense, carbohydrate-rich foods can be a way of self-medicating anti stress and the positive effect of carbohydrates on mood is proportional to the glycemic index of foods. For this reason they could represent the best allies to fight quarantine-related low mood thus giving rise to the so called ‘emotional eating’ [9–11]. This condition is further exacerbated by the disruption in everyday life that along with continuously hearing or reading about the pandemic, could have led to a stressful condition pushing people toward overeating and increasing the risk of developing obesity.

This latter per se is currently one of the most important risk factors of sleep disturbance [3, 12, 13]. Although obesity-related sleep disturbance are mostly due to obstructive sleep apnea (OSA) [14], that is characterized by recurrent narrowing and closure of the upper airway, leading to intermittent oxyhemoglobin desaturation, sleep fragmentation and daytime sleepiness [15], both sleep quality (SQ), and sleep duration have been found to be blunted in subjects with obesity without OSA [16]. Indeed Resta et al. carried out a study in subjects with obesity and without OSA or diseases that are known to be associated with sleep disturbance such as endocrine, psychiatric, and neuromuscular diseases [16]. They found a high prevalence of sleep disturbance such as choking, awakening and unrefreshing sleep, in subjects with obesity than in normal-weight subjects [16]. In addition to OSA, sleep disturbance in obesity could be caused by other obesity-related diseases, such as functional gastrointestinal disorders, including irritable bowel syndrome and functional dyspepsia, nicturia, asthma and osteoarticular pain [12, 17].

In both genders waist circumference has been reported to have a tight association with sleep disturbance [18]. This is because visceral adipose tissue is the main source of pro-inflammatory cytokines, such as interleukin (IL)-1, IL-6 and tumor necrosis factor (TNF)-α that, beyond chronic low-grade inflammation [19, 20], could have a role in sleep regulation such that they are called ‘sleep-regulatory substances’ [21, 22]. Quarantine was also associated with a change of working modalities in most of the people. Indeed they switched to smart-working that could be associated not only to a greater energy intake but also to a decrease secretion of melatonin due to the evening screen time, thus contributing to sabotage sleep [23].

In light of what has been said so far, the primary objective of this study was to investigate the effect of quarantine on sleep quality and on body mass index in Italian adults. The second objective of the study was to investigate if change in sleep quality after quarantine was related to working modalities.

## Materials and methods

### Design and setting

This was a retrospective study carried out at the Department of Clinical Medicine and Surgery, Unit of Endocrinology, University Federico II, Naples (Italy), from January 2020 to 30 April 2020 in accordance with the Code of Ethics of the World Medical Association (Declaration of Helsinki) for experiments involving humans. The purpose of the protocol was explained to all the study participants, and their consent was obtained.

### Population study

All participants started quarantine on March 12th 2020 and the effects on sleep quality were assessed after 40 days. In order to increase the homogeneity of the subject samples, we included only adults of both gender with the following criteria of exclusion evaluated at baseline:

- Clinical conditions such as schizophrenia, depression, chronic or recurrent respiratory conditions, active cancer or neurological disorders;
- Chronic metabolic and cardiovascular diseases including type 2 diabetes mellitus, hypertension, dyslipidemia that could interfere with sleep disturbance;
- Smoking subjects;
- Sleep disorders such as obstructive sleep apnea–hypopnea syndrome;
- Excessive alcohol use (> 4 cups/day) or excessive caffeine use (> 500 mg/day);
- Pregnancy or lactation.

One-hundred twenty-one subjects (43 males and 78 females) aged 18–65 years were found to be eligible.

### Power size justification

The power sample was calculated by the differences of means ± standard deviations (SD) of the Pittsburgh Sleep Quality Index (PSQI) global score at pre and post quarantine (6.37 ± 3.96 vs 8.64 ± 3.73, *p *< 0.001). Considering a type I (alpha) error of 0.05 (95%), and a type II (beta) of 0.05, and the calculated power size was 95%, the minimum number of cases required for a statistical power of 95% was of 40 cases. The calculation of sample size and power were performed while using Sample Size Calculator Clinical Calc (https://clincalc.com/stats/samplesize.aspx), as previously reported in other studies [24–26].

### Data collection and measurements

Data were collected at baseline in our obesity outpatient clinic by face-to-face assessment and after 40 days of quarantine by the telephone interview (self-reported). The phone calls are based on interviewing for specific related questions on anthropometric data, physical activity data, sleep quality, current medications of sleep disturbance and working modalities. For self-measurement of anthropometric data, instructions were given to the participants by a qualified nutritionist.

### Anthropometric measurements

At baseline, measurements were performed between 8 am and 10 am, after an overnight fast. A single nutritionist measured weight and height parameters performed following standard criteria by the same nutritionist according to the International Society for the Advancement of Kinanthropometry (ISAK 2006). The participants were recommended to dress light clothes and to remove shoes during the assessment, as previously reported [27–29]. Body mass index (BMI) [weight (kg) divided by height squared (m^2^), kg/m^2^] was calculated after measuring weight and height. A wall-mounted stadiometer (Seca 711; Seca, Hamburg, Germany) was used to measure height while a calibrated balance beam scale (Seca 711; Seca, Hamburg, Germany) was used to assess weight. The degree of obesity was established according to World Health Organization’s criteria: BMI: 18.5–24.9 kg/m^2^, normal-weight; 25.0–29.9 kg/m^2^, over-weight; BMI: 30.0–34.9 kg/m^2^, grade I obesity; BMI: 35.0–39.9 kg/m^2^, grade II obesity; BMI ≥ 40.0 kg/m^2^, grade III obesity [30].

After 40 days of quarantine, weight and height were collected by telephone interview. Participants were asked to report their body weight with the question: what is your current body weight? (in kg). In particular, self-reported body weight was collected asking the subjects to weight in the morning before breakfast under wearing light clothes (round to 0.5 kg). Self-reported height was obtained asking the subject: “What is your height?” in cm, as also previously reported by Dekkers et al. [31].

### Assessment of sleep quality, physical activity, and working modalities

Sleep quality, physical activity, and working modalities were evaluated during the telephone interview (self-reported). Sleep quality was evaluated using the Pittsburgh Index questionnaire that include seven components: subjective sleep quality, sleep latency, sleep duration, habitual sleep efficiency, sleep disturbances, sleep medication use, and daytime dysfunction [32]. Each of these seven items are equally weighted based on 0 to 3 points, whereby three reflects the negative extreme on the Likert Scale. The global PSQI score ranging from zero to 21 points, with higher scores indicating poorer sleep quality. In according to Buysse DJ et al. poor sleep quality was defined as a PSQI score ≥ 5, while good sleep quality was defined as PSQI score < 5 [32]. The participants habitually engaged in at least 30 min/day of aerobic exercise (YES/NO) was defined as physically active, as we have already fully reported in previous studies [33, 34]. Working modalities have been evaluated as: smart working (YES/NO).

### Statistical analysis

Results are expressed as mean ± SD and categorical variables are expressed as a percentage. Differences between pre and post quarantine were analyzed by Student’s paired *t* test or by Student’s impaired *t* test for the differences between males and females. The Chi square (χ^2^) test was used to determine the significance of differences in frequency distribution of BMI categories, PSQI categories and physical activity. Differences in Δ% variation of PSQI score pre and post-quarantine in the population study across BMI categories (normal weight, over-weight, grade I obesity, grade II obesity and grade III obesity) were analyzed by ANOVA test, with the Bonferroni test as post hoc test. An Alfa α level of 0.05 (type 1 error) and a β level of 0.2 (type II error) were used as the cut-off values for statistical significance. Variables with a variance inflation factor > 10 were excluded in order to avoid multicollinearity. Values ≤ 5% were considered statistically significant. Data were collected and analyzed using the MedCalc^®^ package (Version 12.3.0 1993–2012 -Mariakerke, Belgium).

## Results

All participants started quarantine on March 12th 2020 and were interviewed after 40 days of quarantine by the same operator. Forty-three (35.5%) participants were males, aged 44.9 ± 13.3 years. The characteristics of 121 participants, including anthropometric characteristics and physical activity pre and post quarantine were summarized in Table 1. BMI significantly increased post quarantine (*p *< 0.001) in all cohort and in both genders. Subjects reported to reduce physical activity during the quarantine (*p *= 0.004).

**Table 1:** Anthropometric characteristics and physical activity of the study population pre and post-quarantine

Parameters	Participants pre-quarantine mean ± SD or number (%) n = 121	Participants post-quarantine mean ± SD or number (%) n = 121	*p value
Weight (kg)	88.1 ± 18.9	89.9 ± 19.2	*< 0.001*
Height (m)	1.64 ± 0.1	1.64 ± 0.1	0.516
BMI (kg/m^2^)	32.6 ± 6.0	33.3 ± 6.2	<* 0.001*
Males	33.5 ± 6.1	33.9 ± 5.9	*0.045*
Females	32.1 ± 5.9	32.9 ± 6.3	*0.001*
Normal-weight (n, %)	11, 9.1%	7, 5.8%	χ^2^ = 0.54, *p *= 0.462
Overweight (n, %)	31, 25.6%	30, 24.8%	χ^2^ = 0.01, *p *= 1.000
Grade I obesity (n, %)	41, 33.9%	40, 33.1%	χ^2^ = 0.01, *p *= 1.000
Grade II obesity (n, %)	25, 20.7%	25, 20.7%	χ^2^ = 0.02, *p *= 0.874
Grade III obesity (n, %)	13, 10.7%	19, 15.7%	χ^2^ = 0.90, *p *= 0.342
Physical activity (yes)	62, 51.2%	39, 32.2%	χ^2^ = 8.23, *p *= 0.004

*A *p* value in italic type denotes a significant difference (*p *< 0.05). SD, standard deviation; BMI, Body mass index

Table 2 showed the differences of the single items of PSQI questionnaire, PSQI global score and PSQI categories of the studied population pre and post quarantine. Sleep quality significantly worsened during quarantine as demonstrated by the increase of PSQI score (*p *< 0.001), sleep onset latency score (*p *< 0.001), sleep efficiency score (*p *= 0.003), sleep disturbance score (*p *< 0.001), and daytime dysfunction score (*p *< 0.001). Thirty% of participants worsened their sleep quality (*p *< 0.001).

**Table 2:** Single items of PSQI, PSQI global score and PSQI categories of the study population pre and post-quarantine

Parameters of PSQI questionnarie	Participants pre-quarantine mean ± SD n = 121	Participants post-quarantine mean ± SD n = 121	*p-value
Sleep quality	1.05 ± 0.85	1.69 ± 0.83	*< 0.001*
Sleep onset latency	1.03 ± 0.98	1.61 ± 1.09	*< 0.001*
Sleep duration	1.07 ± 1.04	1.06 ± 0.97	0.902
Sleep efficiency	0.61 ± 0.98	0.88 ± 1.07	*0.003*
Sleep disturbance	1.26 ± 0.68	1.71 ± 0.77	*< 0.001*
Hypnotic drugs	0.31 ± 0.81	0.22 ± 0.69	0.068
Daytime dysfunction	1.04 ± 0.98	1.46 ± 0.96	*< 0.001*
PSQI global score	6.37 ± 3.96	8.64 ± 3.73	*< 0.001*
PSQI categories			
PSQI < 5	60, 49.6%	23, 19.0%	*χ*^*2*^* = 23.76*
PSQI ≥ 5	61, 50.4%	98, 81.0%	*p < 0.001*

*A *p* value in italic type denotes a significant difference (*p *< 0.05)

**Fig. 1:**
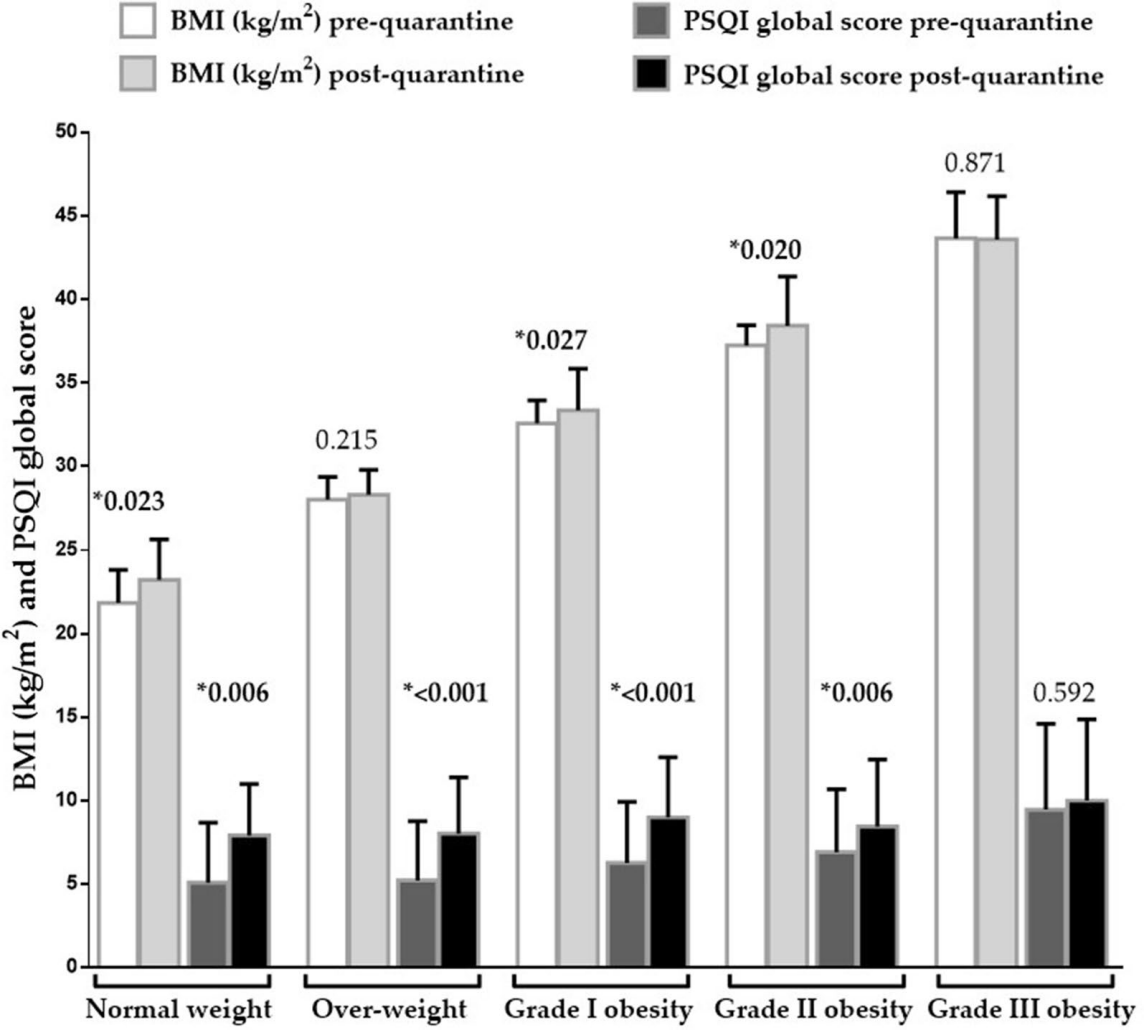
The BMI and PSQI global score pre and post quarantine in the population study across BMI categories. A *p value denotes a significant difference (*p* < 0.05). BMI, body mass index; PSQI, Pittsburgh Sleep Quality Index

The BMI and PSQI global score pre and post-quarantine in the population study across BMI categories were shown in Fig. 1. As reported, stratifying the sample population according to the BMI categories pre and post-quarantine, BMI increased in post-quarantine in normal weight (11 subjects, 21.84 ± 1.98 kg/m^2^ vs 23.22 ± 2.42 kg/m^2^), grade I obesity (41 subjects, 32.58 ± 1.38 kg/m^2^ vs 33.35 ± 2.49 kg/m^2^), and grade II obesity (25 subjects, 37.23 ± 1.21 kg/m^2^ vs 38.41 ± 2.94 kg/m^2^); while no differences were observed in overweight (31 subjects, 28.02 ± 1.34 kg/m^2^ vs 28.30 ± 1.49 kg/m^2^) and grade III obesity (13 subjects, 43.65 ± 2.77 kg/m^2^ vs 43.59 ± 2.59 kg/m^2^). Accordingly in post-quarantine PSQI global score was increased in normal weight (11 subjects, 5.09 ± 3.59 vs 7.91 ± 3.08 score), over weight (31 subjects, 5.22 ± 3.53 vs 8.03 ± 3.37 score), grade I obesity (41 subjects, 6.27 ± 3.66 vs 9.00 ± 3.59 score), and grade II obesity (25 subjects, 6.92 ± 3.76 *vs* 8.44 ± 4.02 score), but no difference has been shown in grade III obesity (13 subjects, 9.46 ± 5.15 vs 10.00 ± 4.86 score); Fig. 1.

**Fig. 2:**
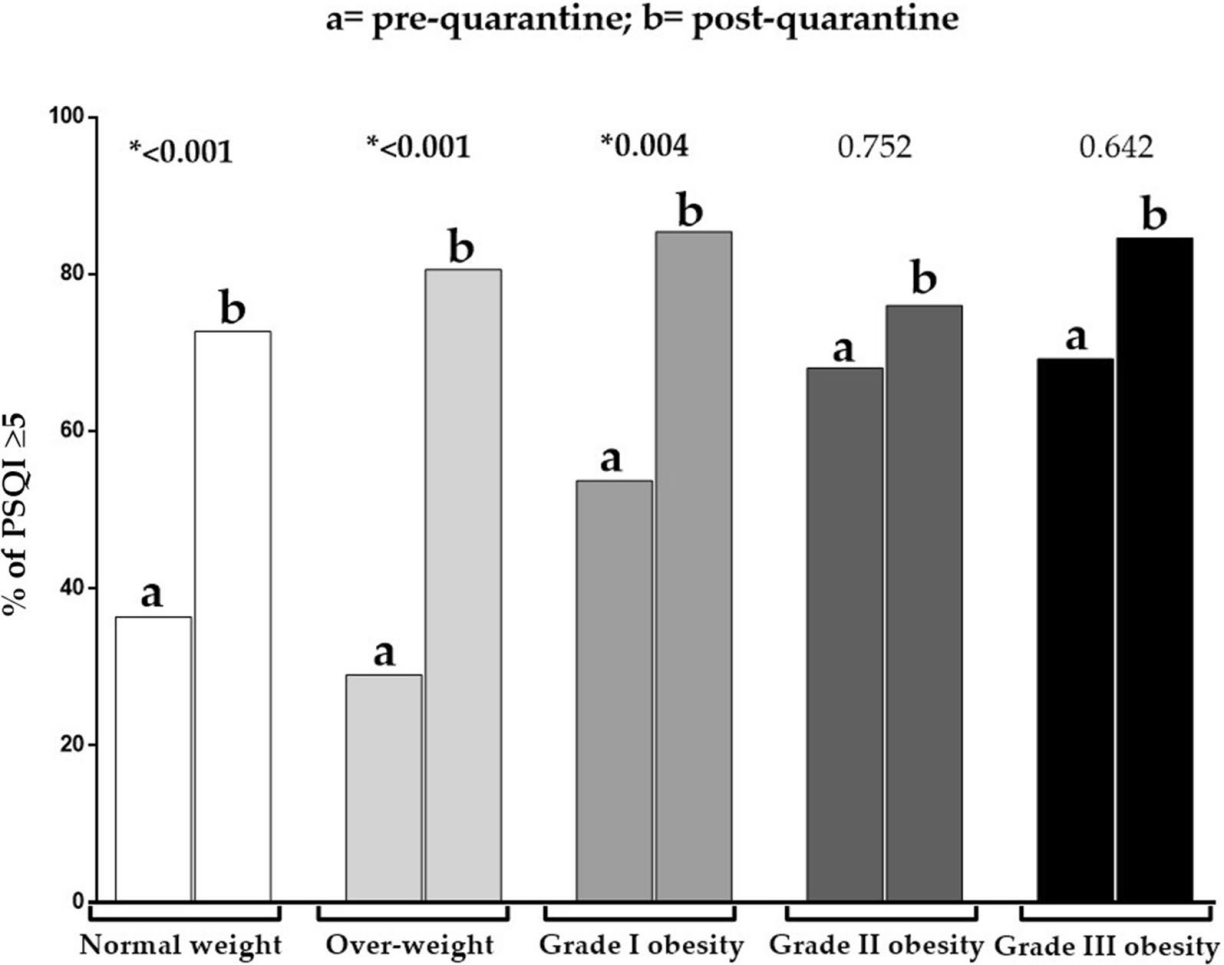
Percentage of PSQI ≥ 5 pre and post-quarantine across BMI categories. A *p value denotes a significant difference (*p* < 0.05). PSQI, Pittsburgh Sleep Quality Index

Figure 2 reported the % of poor sleepers (PSQI ≥ 5) pre and post-quarantine across BMI categories. In detail, stratifying the sample population according to BMI categories, the % of poor sleepers significantly increases in normal weight (36.4% vs 72.7%), over weight (29% vs 80.6%) and grade I obesity (53.7% vs 85.4%); on the contrary, no difference was observed in grade II obesity (68.0% vs 76.0%) and grade III obesity (69.2% vs 84.6%); Fig. 2.

**Fig. 3:**
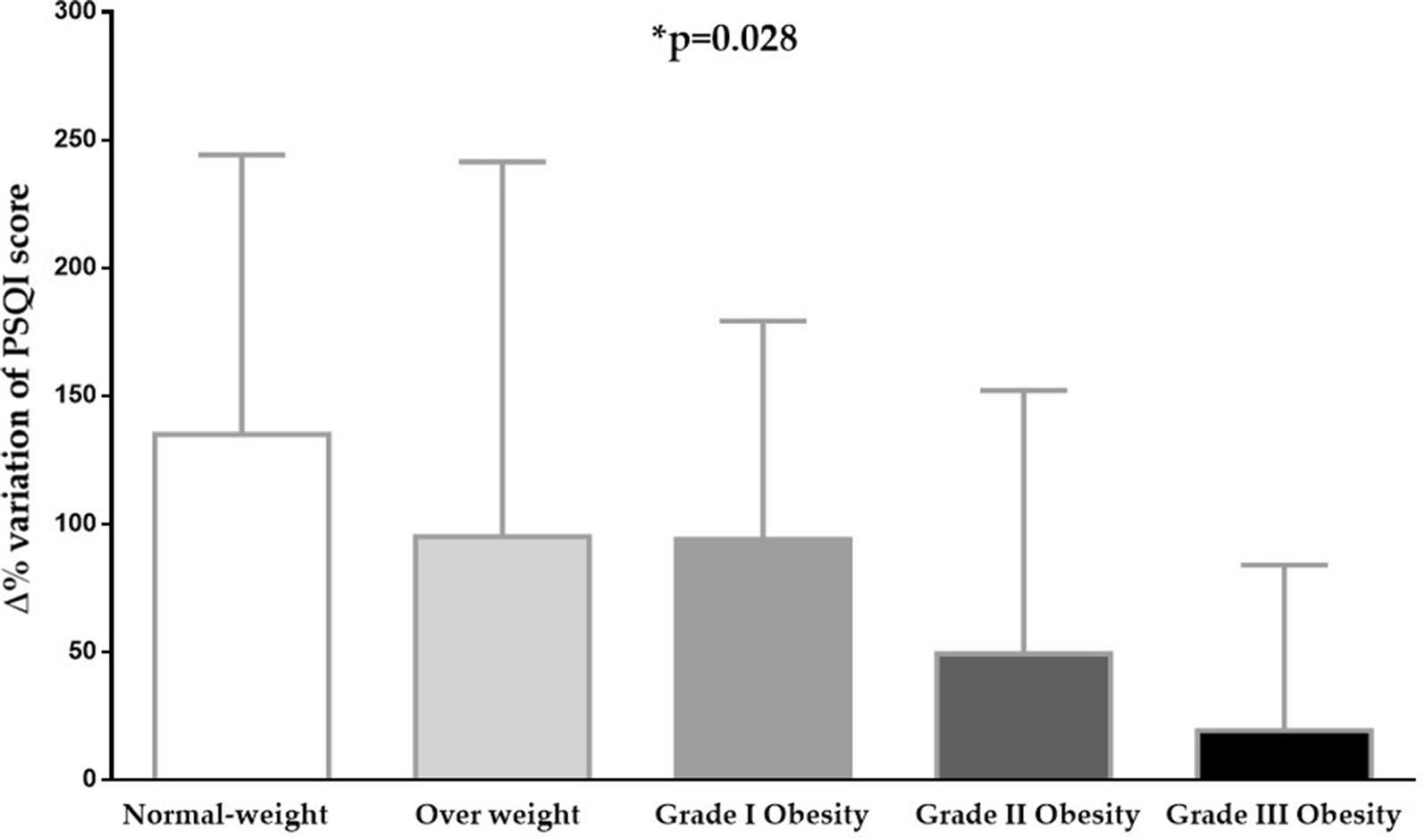
Delta % variation of PSQI score pre and post-quarantine in the population study across BMI categories. A *p value denotes a significant difference (*p* < 0.05). PSQI, Pittsburgh Sleep Quality Index

The Δ% variation of PSQI score pre and post-quarantine in the population study across BMI categories were shown in Fig. 3. As showed, stratifying the sample population across the BMI categories the Δ% variation of PSQI score decreases with increasing BMI categories. In particular, normal weight had the highest Δ% variation of PSQI score (135.00 ± 109.16) compared to overweight (95.16 ± 146.35), grade I obesity (94.05 ± 85.14), grade II obesity (49.43 ± 102.71), and grade II obesity (19.29 ± 64.66); Fig. 3.

The pre and post quarantine gender difference in the single items of PSQI questionnaire, PSQI global score and PSQI categories of the study population, were reported in Table 3. In both gender, there was a worsening of sleep quality. Of interest, although at baseline the percentage of good sleepers were higher in males than females (60.5% vs 43.6% of PSQI < 5), a higher percentage of males became poor sleepers compared to females (44.2% vs 23.1% of PSQI ≥ 5). In addition, a higher percentage of males decreased physical activity levels (− 27.9%, *p *= 0.015), while no significant differences were shown in females (*p *= 0.106). However, a gender difference in terms of changes of physical activity habits was reported post quarantine (*p *= 0.016) (Table 3).

**Table 3:** Single items of PSQI, PSQI global score and PSQI categories of the study population pre and post quarantine, according to gender

Parameters	Male participants mean ± SD n = 43				Female participants mean ± SD n = 78				
	Pre quarantine	Post quarantine	Δ%	*p-value	Pre quarantine	Post quarantine	Δ %	*p-value	**p-value Δ%
Sleep quality	0.91 ± 0.94	1.49 ± 0.73	48.83 ± 58.99	*< 0.001*	1.13 ± 0.77	1.81 ± 0.85	47.22 ± 72.49	*< 0.001*	0.901
Sleep onset latency	0.98 ± 1.06	1.58 ± 1.00	54.45 ± 76.29	*<0.001*	1.06 ± 0.94	1.63 ± 1.14	48.50 ± 90.10	*<0.001*	0.701
Sleep duration	1.11 ± 1.16	1.02 ± 1.10	2.33 ± 33.10	0.400	1.04 ± 0.97	1.08 ± 0.89	11.75 ± 49.50	0.650	0.214
Sleep efficiency	0.56 ± 0.96	0.72 ± 0.91	19.96 ± 43.54	0.109	0.64 ± 1.00	0.97 ± 1.14	41.24 ± 94.30	*0.011*	0.093
Sleep disturbance	1.11 ± 0.66	1.77 ± 0.81	40.31 ± 56.84	*< 0.001*	1.35 ± 0.68	1.68 ± 0.75	20.94 ± 43.16	*0.002*	*0.037*
Hypnotic drugs	0.21 ± 0.63	0.21 ± 0.63	0.00 ± 0.00	0.999	0.36 ± 0.89	0.23 ± 0.72	-2.88 ± 20.13	0.068	0.350
Daytime dysfunction	0.79 ± 0.89	1.51 ± 0.86	60.27 ± 67.50	*< 0.001*	1.18 ± 1.01	1.44 ± 1.01	35.89 ± 82.98	0.084	0.083
PSQI global score	5.67 ± 4.31	8.30 ± 3.63	98.85 ± 103.60	*< 0.001*	6.76 ± 3.73	8.83 ± 3.80	62.99 ± 110.75	*< 0.001*	0.079
PSQI categories									
PSQI < 5	26, 60.5%	7, 16.3%	χ^2^ = 15.93 *p *= 0.001		34, 43.6%	16, 20.5%	χ^2^ = 8.51 *p *= 0.004		χ^2^ = 28.82 *p *< 0.001
PSQI ≥ 5	17, 39.5%	36, 83.7%			44, 56.4%	62, 79.5%			
Physical activity									
Yes	23, 53.5%	11, 25.6%	χ^2^ = 5.88 *p *= 0.015		39, 50.0%	28, 35.9%	χ^2^ = 2.62 *p *= 0.106		χ^2^ = 10.34 *p *= 0.016
No	20, 46.5%	32, 74.4%			39, 50.0%	50, 64.1%			

*A *p* value in italic type denotes a significant difference (*p *< 0.05) within the group between pre and post quarantine. **A *p* value in italic type denotes a significant difference (*p *< 0.05) of Δ% of variation between males and females

The differences in Δ% variation of PSQI score according to working modalities in all participants divided by gender, were summarized in Table 4. In both males (*p *< 0.001) and females (*p *= 0.002) there was a worsening of sleep quality that was significantly higher in subjects performing smart working compared to subjects not performing smart working. In addition, males performing smart working had a significantly higher worsening of sleep quality than females performing smart working (*p *< 0.001).

**Table 4:** Differences in ΔPSQI score according to working modalities in all participants divided by gender

Parameters	All participants n = 121	n	Male participants n = 43	n	Female participants n = 78	**p-value
Smart working						
Yes	39, 32.2%	16	151.41 ± 94.33	23	87.29 ± 115.52	*< 0.001*
No	82, 67.8%	27	10.17 ± 35.61	55	4.92 ± 71.96	*0.027*
*p*-value			*p < 0.001		*p = 0.002	

*A *p* value in italic type denotes a significant difference (*p *< 0.05) in Δ% variation of PSQI in smart working mode (yes vs no) in males and females. **A *p* value in italic type denotes a significant difference (*p *< 0.05) of Δ % variation of PSQI between males and females

## Discussion

To the best of our knowledge, this study was the first to investigate the effect of quarantine/lockdown due to the COVID-19 pandemic on sleep quality in Italian population subjects. The main results of our study are the worsening sleep quality, the increase in BMI and the reduction of physical activity after quarantine. Sleep quality was mostly worsened in subjects performing smart working compared to subjects not performing smart working in both genders. Males performing smart working had a significantly worse sleep quality than their female counterparts working in the same modality.

### Sleep quality

Study findings revealed that quarantine resulted in a worsening of sleep quality in normal weight and overweight subjects and in subjects with grade I and II obesity. In particular, there was an increase in sleep onset latency and sleep disturbance in both males and females. There was a significant worsening of sleep efficiency in females while there was a significant worsening of daytime dysfunction in males. There was an increase in BMI in normal weight and in subjects with grade I and II obesity after 40 days of quarantine. In addition, physical activity significantly decreased in all cohort of subjects. However, females had a trend toward the decrease of physical activity while males significantly decreased physical activity. As well known, on March 12th 2020 Italian people experienced locked down in order to reduce the widespread of the pandemic of coronavirus. As consequence, quarantine was associated to the interruption of work routine and these results in economic issues due to economic activity stalls and job losses mount. This generates worries about income, savings, and making ends meet. As a consequence of the lockdown, trips were canceled, and people were isolated from friends and family. Therefore, the isolation at home could generate the depression or exacerbate it in people that were already affected. Along with these situations, continuously hearing or reading about the pandemic without a break contributes to the onset of stress. As well known, stress is an adaptive response of the organism to real or perceived stressors. The main components of the stress system are the corticotropin-releasing hormone and locus coeruleus-norepinephrine/autonomic systems that are connected to peripheral effectors such as the hypothalamic–pituitary–adrenal axis, and the limbs of the autonomic system [35]. It has been reported that hypercortisolism can lead to fragmentation of sleep, decreased slow-wave sleep, and shortened sleep time. To complicate matters, sleep disturbances can in turn further worsen hypercortisolism and thereby worsening the cycle [36]. Interestingly the worsening of sleep quality was more evident in subjects with normal weight and in subjects with grade I and II obesity. No further worsening was noticed in subjects with obesity grade III. This could be because subjects with obesity III grade had already a poor sleep quality at the baseline and there was not further worsening margin. As well known, the degree of obesity is directly correlated with sleep quality [12]. Subjects with normal weight and obesity I and II grade experienced an increase of their BMI. This could be explained because they reduced physical activity. Indeed, the excess of adipose tissue narrows breath airways but it is also involved in the releases of cytokines [37]. In particular IL-1, IL-6 and TNF-α are not only pro-inflammatory cytokines but also a ‘sleep-regulatory substances’ [21, 38]. TNF-α and IL-1b, whose secretion follows a circadian rhythm, with the highest TNF-α and IL-6 secretion during the night (between 01:00 and 02:00 h), are involved in the physiological regulation of sleep in both animals and humans [39], playing an important role in the slow-wave sleep [40]. Interestingly males reported a decrease in physical activity. Accordingly, it has been reported that during quarantine outdoor activities and social activities are prohibited by the governments and this could result in a decrease of physical activity [41]. Thus, a consequence of quarantine is limited physical activity that in turn results in an increased risk of developing obesity. On the contrary, previous studies reported that males are more active than females in leisure-time [42, 43]. Indeed Martin- Gonzalez et al. performed a study investigating the prevalence of physical activity during leisure time in adults from the 15 member states of the European Union and the relationship with socio demographic variables finding that a higher percentage of males practiced leisure-time physical activity [42]. Similar results were found by Steptoe et al. that carried out a survey in university students from 13 European countries (Belgium, England, France, Germany, Greece, Hungary, Iceland, Ireland, Italy, The Netherlands, Poland, Portugal, and Spain) in 1990 and repeated in 2000 highlighting that the prevalence of leisure-time physical activity was higher in males [43]. Another aspect to be considered regards the diet that is known to affect both body weight and sleep quality [4, 44].

Evidence demonstrated that a higher energy intake notably from fat [45] and snacks [46], has been frequently reported in poor sleepers than in normal sleepers. NHANES data reported that poor sleepers compared to normal sleepers (7–8 h of sleep/night) had a lower consumption of protein, fat, carbohydrate, and fiber [47]. Clinical intervention studies have corroborated these evidences reporting that during sleep restriction fat was also highlighted as a macronutrient of choice in subjects with a normal habitual sleep [48, 49]. Although studies reported that there is a relationship between sleep quality and diet these epidemiologic evidence cannot address causality or the direction of the relation among these variables. In fact, it is unknown whether it is dietary intake that affects or sleep quality, or *viceversa.* Relationship between dietary patterns and sleep quality were recently showed in a cross-sectional study [50]. In this study, a high intake of fresh food like vegetables and fish, were associated with good sleep quality, whereas a high consumption of packaged and processed foods such as confectionary were associated with poor sleep quality, evaluated by a high global Pittsburgh Sleep Quality Index score [50]. Also a poor sleep quality was evident in subjects who consumed sugar-sweetened beverages or energy drinks [50]. In addition, other epidemiologic studies have found the relationship between diet and sleep quality [51–53], highlighting that both low and high intake of protein (< 16% and > 19% of energy from protein, respectively) were associated with poor sleep quality characterized by difficulty in maintaining sleep. Some foods, especially fresh ones, including fish, fruit, vegetables, and milk products have been identified as sleep-inducers; however, longer-term effects of dietary intake on sleep quality have not been examined in randomized controlled studies [4, 44].

Even if not evaluated in this study, we hypothesize that a change in eating habits due to increased hunger and decreased satiety could contribute to weight gain observed in our studied population. Another important aspect to consider regarding diet is that during quarantine/lockdown, the individuals could have spent more time than usually happens in the kitchen for cook, with a consequent increase in the intake of carbohydrate, mostly pizza, homemade desserts and bread. In fact, as reported in the paragraph above, there is a bidirectional relationship between diet-obesity and sleep disturbance. Finally the house confinement imposed by quarantine could be associated to the decreased intake of fresh food, as fruit, vegetables and fish, all food rich in vitamins (including vitamin C, beta-carotene, and vitamin D), minerals (including selenium and zinc) and omega 3 fatty acids with anti-inflammatory and antioxidants activities. The low consumption of these micronutrients is associated with both obesity and sleep disturbance [54–56]; in particular, vitamin D plays a key role in the relationship between obesity and sleep disturbances [57–60].

### Working modalities

Another interesting finding of our study is that smart working was associated to a greater deterioration of sleep quality that was more pronounced in males than females. This could be due to smart-device overuse and it is in agreement with a previous study carried out in 494 participants, from the three major cities in the United Arab Emirates finding that 81% of poor sleepers were heavy users of smart-devices thus concluding that poor sleep is strongly correlated with smart-device overuse [61]. In particular, poor sleepers have been reported to be five times more likely to be overuses [61]. Further, subjects in quarantine could be lead to check the news on phone, to join a virtual meeting with family or friends, to watch TV, or to stay at computer later in the evening thus resulting in a huge increase in screen time. Excess screen time, especially later in the evening, can have a detrimental impact on sleep. Not only it can stimulate the brain in ways that makes it hard to wind down, but the blue light from screens can suppress the natural production of melatonin, a hormone that is known to be the main key player of sleep [62, 63].

### Limits and strengths

We are aware that there are some limitations in the current study. First, the main limitation of this study is represented by a self-reported weight after quarantine. However, other studies have carried out the same evaluation modality [31]. Second, the sample size was relatively small. Nevertheless, we have calculated the sample size using 95% power. The number of cases required was 40, while we used 121 individuals i.e. more than double those required. Third, although it is well known that dietary intake are important determinants of both weight gain and sleep disturbance, we did not include in this study the diet analysis.

However, the strengths of this study are several. In particular, this study provide unique information regarding a particular social condition represented by quarantine during a pandemic. Nevertheless, a major strength of this study is the homogeneity of our sample population that further strengthens the power of the study. In particular we have adopted stringent exclusion criteria including clinical conditions which could interfere with sleep quality like neurological disorders, chronic metabolic and cardiovascular diseases, and smoking subjects, known factors that can affect both sleep quality and weight gain making it possible to compare the variables independently across subjects. In addition, all study individuals lived in the same geographical area, Naples metropolitan area (latitude 40°49′ N; elevation 17 m), likely with the similar nutrient availability and food consumption patterns, which allowed us to increase the homogeneity of the study sample. Furthermore, we included a variety of potential covariates, such gender and smart working, to minimize the effect of confounding factors on the role of quarantine/lockdown on sleep quality. An additional strength is the Pittsburg Sleep Quality Index questionnaire that was by phone administered and not self-reported in order to minimize any bias related to the filling of the questionnaire. To avoid inter-operator variability, only four expert nutritionists administered and calculated the Pittsburg Sleep Quality Index questionnaire and the telephone interview at the baseline and at the follow up.

## Conclusions

To the best of our knowledge, this study was the first to investigate the acute impact of quarantine/lockdown due to the COVID-19 pandemic on sleep quality modification, after 40 days of quarantine/lockdown. In summary, our study demonstrated that quarantine was associated to a significant increase in BMI and a decrease in sleep quality. In particular, there was a worsening of sleep onset latency, sleep efficiency, sleep disturbances and daytime dysfunction. The worsening of sleep quality was higher in subjects doing smart working and this finding was more pronounced in males. Therefore, during quarantine it could be advisable to adopt lifestyle strategies in order to counteract quarantine-related sleep disturbances such as consuming food containing or promoting the synthesis of serotonin and melatonin at dinner such as roots, leaves, fruits, and seeds such as almonds, bananas, cherries, and oats and increasing physical activity.

## Data Availability

All data generated or analyzed during this study are included in this published article.

## Abbreviations

SARS-CoV-2: Severe acute respiratory syndrome coronavirus 2
OSA: Obstructive sleep apnea
SQ: Sleep quality
IL: Interleukin
TNF: Tumor necrosis factor
SD: Standard deviations
PSQI: Pittsburgh sleep quality index
BMI: Body mass index
